# The Role of Musculoskeletal Ultrasound in the Rheumatoid Arthritis Continuum

**DOI:** 10.1007/s11926-020-00911-w

**Published:** 2020-06-19

**Authors:** Andrea Di Matteo, Kulveer Mankia, Masayuki Azukizawa, Richard J Wakefield

**Affiliations:** 1 Leeds Institute of Rheumatic and Musculoskeletal Medicine, University of Leeds, Leeds, UK; 2 National Institute for Health Research Leeds Biomedical Research Centre, Leeds Teaching Hospitals NHS Trust, Leeds, UK; 3 Department of Clinical and Molecular Sciences, Polytechnic University of Marche, Rheumatology Unit, Carlo Urbani Hospital, Jesi, Ancona, Italy; 4 Department of Orthopaedic Surgery, Kyoto University Graduate School of Medicine, 54 Shogoin Kawahara-cho, Sakyo-ku, Kyoto, 606-8507 Japan; 5 Department of Orthopaedic Surgery, Himeji Medical Center, 68 Honmachi, Himeji, Hyogo 670-8520 Japan

**Keywords:** Musculoskeletal ultrasound, Rheumatoid arthritis, Differential diagnosis, Disease monitoring, Remission

## Abstract

**Purpose of Review:**

Rheumatoid arthritis (RA) is no longer considered a fixed phenotype but rather a disease continuum. This review outlines the current and potential value of applying ultrasound (US) along this continuum: from the prediction of progression to RA in at-risk individuals, to confirmation of the early diagnosis of RA, as well as the consideration of differential diagnoses, and the use in disease monitoring and defining remission.

**Recent Findings:**

In individuals at-risk of RA (i.e., positive autoantibodies with symptoms but without synovitis), US has shown a promising predictive value for the development of clinical arthritis, providing the opportunity to improve risk stratification (and disease prevention) of these individuals. The detection of inflammation on US in patients with early undifferentiated arthritis, in which a definite diagnosis cannot be reached, could predict evolution to persistent arthritis, mostly RA. This, in addition to the US potential ability to identify disease specific patterns for different rheumatic conditions, might facilitate early diagnosis and, therefore, improve the management of patients with RA, or other types of inflammatory arthritides. US has also demonstrated the capability to predict radiographic progression, and relapse risk after treatment discontinuation, in RA patients in remission according to the clinical instruments, raising implications in the management, including therapy discontinuation, of these patients.

**Summary:**

US has an undeniable value in the management of patients at different stages along the RA continuum. Further research is needed to identify which groups of patients benefit the most from US imaging.

## Introduction

### Ultrasound in Rheumatoid Arthritis

In 1997, at the American College of Rheumatology (ACR) pre-course conference, an eminent musculoskeletal radiologist discussed the role of imaging techniques for musculoskeletal diseases. One of the questions asked at the end was ‘What about ultrasound, you didn’t mention it?’ The answer was ‘Well, it is only really useful for Baker’s cysts!’ Coincidently, that year saw the first international trial of Remicade (infliximab) in rheumatoid arthritis (RA), the beginning of the concept of early diagnosis and ‘window of opportunity’, and the launch of a new wave of ultrasound (US) machines which were better adapted for the assessment of musculoskeletal diseases. From this point, there began an increasing rise in the use of musculoskeletal US in rheumatology practice, facilitated through a coordinated approach of education led by the European League Against Rheumatology (EULAR) and the ACR, as well as other national societies [1]. Some countries were swift to embrace the US concept and incorporate it into their educational programmes for new trainees, whilst others have been more cautious, adopting a more ‘wait and watch’, evidence-based approach. Without doubt, the availability of US to rheumatologists was initially met with much anticipation as it provided a direct way of improving the accuracy of physical examination, enabling a deeper understanding of joint pathophysiology, as well as providing a means of guiding needles for interventions. As it was a technique that rheumatologists could potentially perform themselves, it could also enable immediate decision-making and therefore improve efficiency. Over time, falling costs, the development of educational opportunities, and increased credibility as a consequence of expanding experience and evidence base have further facilitated its uptake.

US images from 20 years ago are barely recognizable when compared to those of today. Improvements in image resolution through the greater processing capabilities of computers and the development of higher-frequency transducers employing more sensitive Doppler modalities now enable the depiction of tiny anatomical details (< 0.1 mm resolution) and blood flow. Like with US, much excitement was initially also directed at other advanced imaging techniques, such as magnetic resonance imaging (MRI) and computed tomography (CT) for early disease detection. MRI theoretically appeared the perfect tool allowing simultaneous tomographic imaging of bone and soft tissue. However, despite more recent exploration into whole body MRI techniques, MRI has never gained universal acceptance as a routine imaging technique for RA, largely due to the feasibility aspects, such as availability, cost, and patient tolerance. Many would argue that MRI therefore remains a second/third line imaging tool (after X-ray and US) for equivocal or uncertain cases and second line in axial scanning (after X-ray). In contrast, CT is hampered by its inability to image soft tissue and need for ionizing radiation although it is arguably the best at depicting bone integrity.

**Fig. 1:**
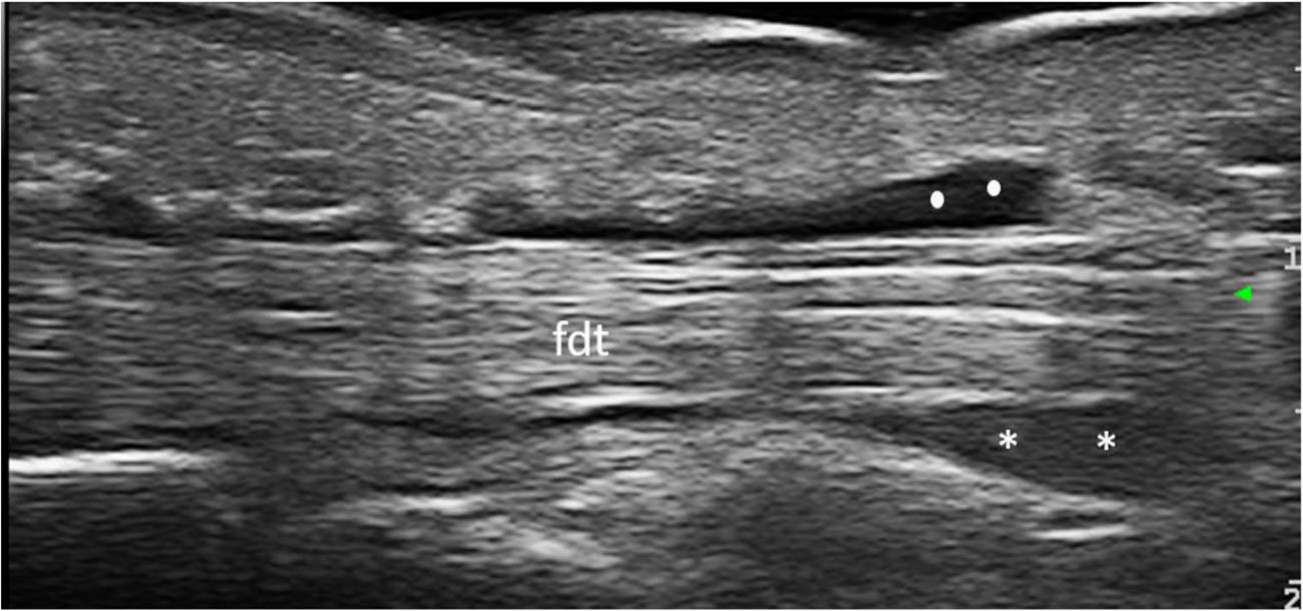
Tenosynovitis of the third flexor digitorum tendons in a patient with rheumatoid arthritis. The longitudinal scan of the flexor digitorum tendons shows the presence of synovial hypertrophy (asterisks) and synovial effusion (rounded dots) in the synovial tendon sheath. Legend: fdt, flexor digitorum tendons

**Fig. 2:**
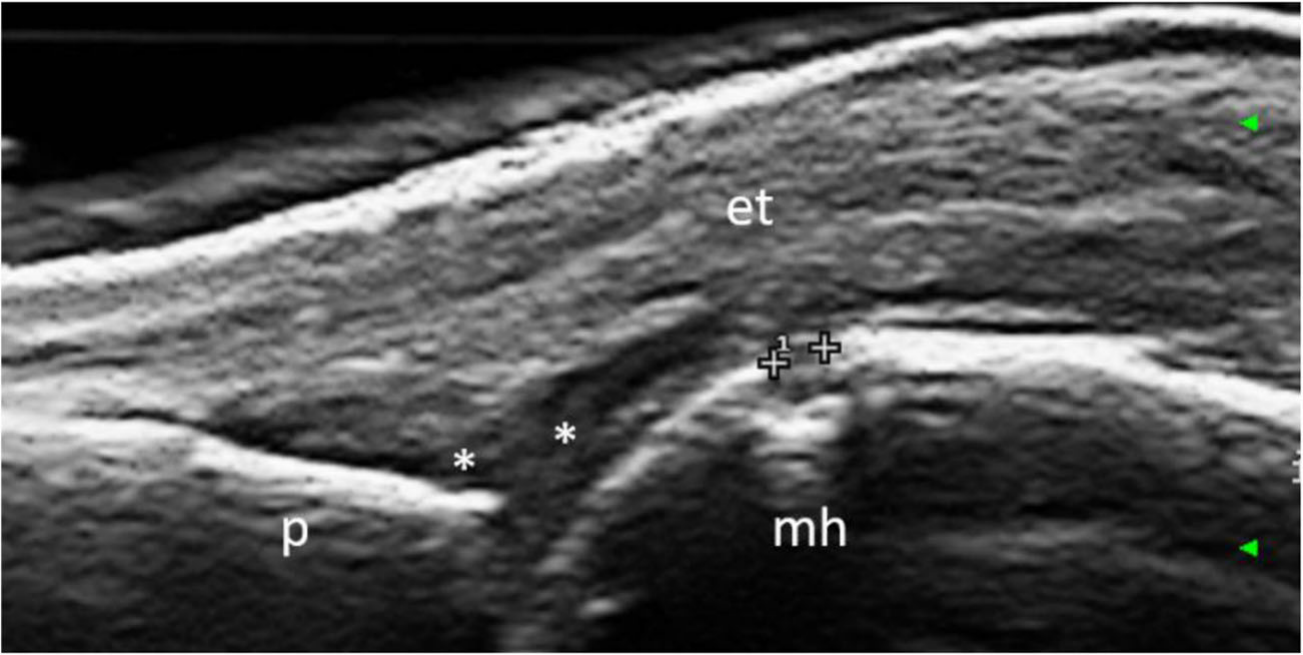
Bone erosion in the second metacarpophalangeal joint in a patient with rheumatoid arthritis. Longitudinal scan. The callipers point out a small bone erosion (size 0.77 mm) in the metacarpal head. Legend: asterisks, synovial hypertrophy; et, extensor tendon; mh, metacarpal head; p, proximal phalanx

In the context of RA, US is able to detect the signs of acute inflammation, such as synovial and tenosynovial effusion (Fig. 1), synovial hypertrophy, power Doppler (PD) signal, or soft tissue oedema, as well as structural damage including bone erosions (Fig. 2), loss of cartilage, or tendon tears [2, 3••].

US offers the opportunity to compare in ‘real-time’ the anatomical findings with clinical assessment. In the evaluation of patients with regional pain, the integration of the US information with the clinical data yields obvious advantages to the rheumatologist.

**Fig. 3:**
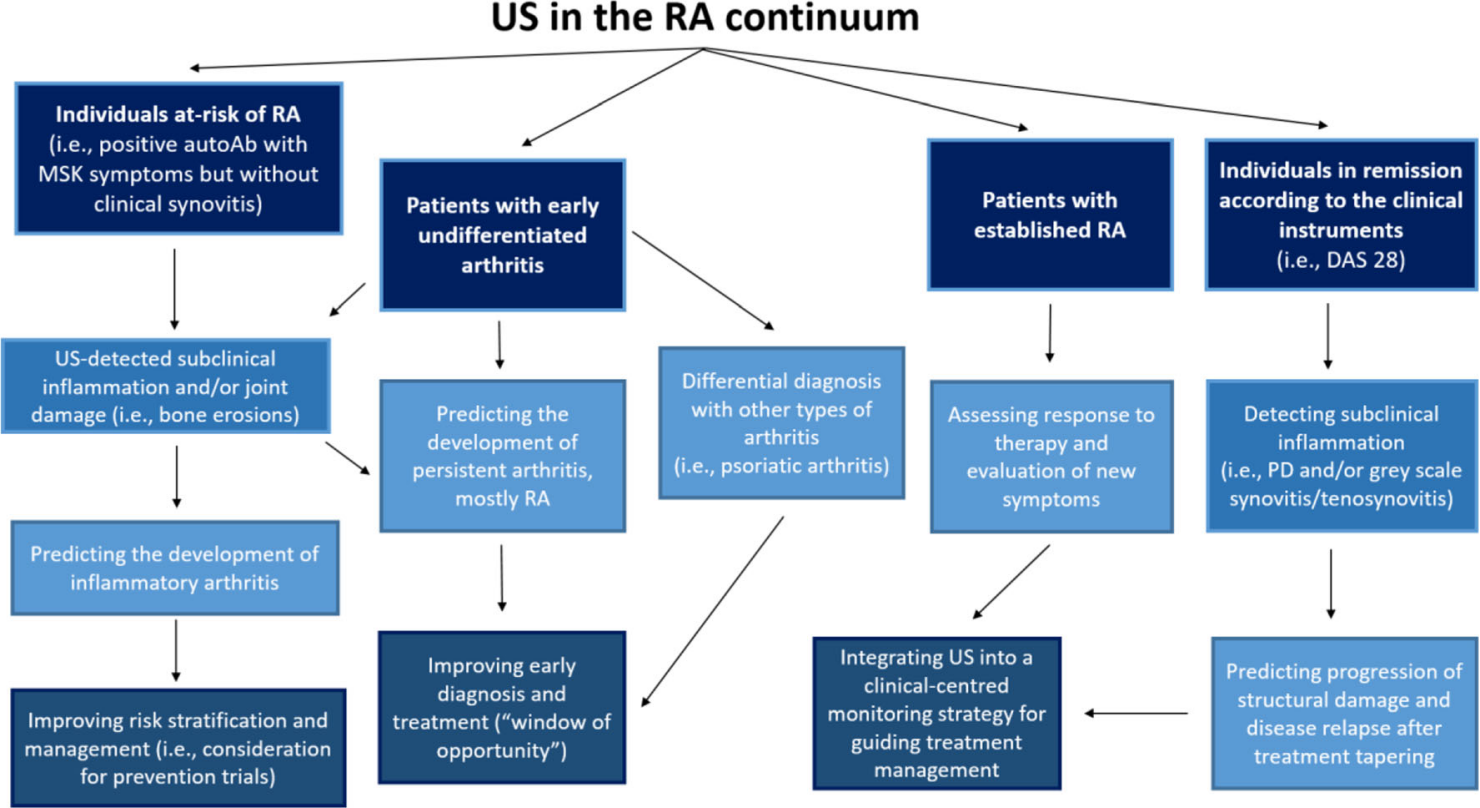
The potential uses of ultrasound in the rheumatoid arthritis continuum. The figure illustrates the potential value of US in the management of patients at different stages along the RA continuum: in individuals at risk of RA, the detection of subclinical synovitis and structural damage has shown to improve prediction of clinical arthritis, thus informing risk stratification and management of these individuals. US has also shown a promising role in the identification of patients with undifferentiated arthritis who will develop RA, with potential implications on early diagnosis and management of these patients (‘window of opportunity’). In RA patients with established disease, in clinical remission according to the clinical instrument (i.e., DAS 28), the detection subclinical inflammation has been shown to predict progression to structural damage and disease relapse after tapering of the treatment. In this context, US has a promising role in guiding the management of these patients, including treatment discontinuation, in addition to the routinely used clinical instruments. Legend: Ab, antibodies; DAS, disease activity score; PD, power Doppler; MSK, musculoskeletal; RA, rheumatoid arthritis; US, ultrasound

This review will highlight how the concept of RA has recently changed in that it should no longer be considered as a fixed phenotype, but one that evolves through different stages of a continuum [4]. The potential utility of US in the management of patients at different stages of this continuum (Fig. 3) will be discussed: predicting progression to RA in at-risk individuals, early diagnosis of RA, differential diagnosis, monitoring, and remission. The authors will attempt to provide a balanced argument of the use of US although their disclosure is that they all use US in their daily clinical practice and are involved in research.

### The Utility of Ultrasound in Managing Individuals at-Risk of Rheumatoid Arthritis without Clinical Synovitis

A major recent advance in rheumatology has been the better understanding of the preclinical phase of RA (‘pre-RA’). This refers to patients who are considered ‘at-risk’ of developing RA but as yet have not developed clinical synovitis or at least enough synovitis to be confident of the diagnosis. In the literature, ‘at-risk’ particularly refers to those patients who have positive autoantibodies with symptoms, such as fatigue and ‘clinically suspect arthralgia’ (CSA) but without signs of clinically detectable inflammation. The potential to identify patients ‘at-risk’ for the future development of RA raises the opportunity to prevent disease progression in these individuals [5]. Many patients will have underlying genetic and environmental predispositions (e.g., an affected first-degree relative and/or cigarette smoking) although this is not necessarily the case. Individuals with autoantibodies and symptoms but no clinical synovitis are currently being followed within ‘at-risk’ cohorts [6, 7] but on an individual basis we need to know which ones will develop clinical synovitis. In the absence of any guidelines, management of these symptomatic at-risk patients is challenging and consequently current practice is highly variable [8]. Both over-treatment and under-treatment will therefore naturally ensue, according to the clinical intuition of the rheumatologist.

In recent years, as the concept of ‘at-risk of RA’ has evolved, biomarkers such as US have been investigated to address two broad areas. First, to improve our understanding of disease pathogenesis, and second, for risk prediction and stratification for intervention. Most rheumatology textbooks inform us that RA is a synovial-based disease. However, two recent studies have implicated the flexor and interosseous finger tendons [9, 10]. This is reinforced by a clinical observational study by Stack et al. which demonstrates that early RA-related inflammation can occur outside the joint capsule, as evidenced by redness and oedema of the skin prior to the onset of RA disease [11]. The involvement of these extra-articular structures might potentially explain the prodrome of non-specific pain and stiffness that may precede clinical synovitis.

Together with clinical and serological biomarkers, US-detected subclinical inflammation and joint damage (i.e., bone erosions) have been added as variables in risk prediction tools for anti-cyclic citrullinated peptide (CCP)-positive individuals at risk of RA [12, 13, 14•]. These tools enable at-risk individuals to be stratified according to risk of inflammatory arthritis (IA) development. In this way, individuals with a low risk of progression can be reassured and monitored, whereas those with a high risk of imminent arthritis can be identified for closer monitoring and potential clinical trials [15].

In a cohort of anti-CCP-positive individuals with musculoskeletal symptoms but no clinical synovitis, the presence of subclinical inflammation on US was strongly associated with the development of IA, both at joint and patient level [12, 16]. The presence of intra-articular PD signal appears to be particularly predictive in these patients; those with a PD score of ≥ 2 in any joint were at significantly higher risk of progression than those without PD (median 7.1 months vs 52.4 months, HR = 3.7, *p* < 0.001). Similarly, individual joints with PD score ≥ 2 were much more likely to develop clinical synovitis (HR 31.3, *p* < 0.001). Patients with an erosion in at least one joint were also more likely to progress to IA than individuals without any erosions (median 7.5 months vs 50.1 months, HR = 2.9, *p* < 0.001) [16]. US findings have also been shown to be associated with IA development in another seropositive arthralgia cohort [17]. In this cohort, 49/163 (30%) of patients had US abnormalities in at least one joint. Although PD was infrequently identified, US detected synovial thickening was associated with development and timing of IA at patient level (median 23 months vs 45 months, HR = 3.4, *p* < 0.01).

Pragmatically, US can also be used to confirm clinical synovitis (and tenosynovitis) in at-risk individuals who are suspected of having progressed to IA. Such patients, in the earliest phases of clinical disease, are often difficult to assess and the clinical findings can be subtle. Clinical trials investigating arthritis prevention in individuals at-risk of RA are increasingly stipulating US confirmation of clinical synovitis for this reason [18].

Despite the several advantages described above, there are some important considerations which must be borne in mind for the optimum use of US in individuals at-risk of RA without clinical synovitis. The points raised below argue for careful and considerate, rather than indiscriminate, use of US in at-risk populations.

First, the subclinical inflammation detectable by US is likely to be a late feature in the development of IA, and when present may represent a risk of imminent clinical synovitis. Serial US assessments in a cohort of anti-CCP-positive at-risk individuals suggest subclinical inflammation develops directly before clinical synovitis occurs [19]. The corollary is that US may not be as informative in lower-risk individuals who do not yet have imminent clinical arthritis. In line with this, a recent US study of 273 first-degree relatives of RA patients (FDRs), of whom only 8% were anti-cyclic citrullinated peptide antibodies (ACPA)-positive, found no overall increase in US synovitis in this population [20]. Likewise, in the Amsterdam seropositive arthralgia cohort, whilst grey-scale synovitis was associated with progression to IA, intra-articular PD signal was infrequently identified and was not predictive of progression to IA [17]. This contrasts with the data from the Leeds CCP cohort, where PD signal was identified in 30% of patients and was strongly associated with development of IA and its timing, both at patient and joint levels [16]. The apparent disparity between the two studies may be due to the different risk profiles of the at-risk individuals. The Leeds cohort are all anti-CCP-positive and include higher-risk subjects, many of whom had imminent IA; 57/136 (42%) developed IA at median 8.6 months. The Amsterdam seropositive arthralgia cohort had comparatively lower-risk subjects; not all are anti-CCP-positive and 51/163 (31%) developed IA at median 12 months. Moreover, when a previously published clinical prediction rule was applied to the Amsterdam cohort [7], the predictive capacity of US findings was observed to be highest in the groups with intermediate and high risk of IA. The authors thus proposed that US may be of most value in these higher-risk patients rather than low-risk individuals [17].

Second, given the now widespread availability of US in early arthritis clinics, it is frequently used to aid diagnosis and guide the management of patients suspected of having IA. Algorithms to guide rheumatologists suggest US should be used to guide management of ACPA-positive individuals with inflammatory joint symptoms without clinical synovitis [21]. In the absence of trial evidence, the optimum management of these patients is not yet clear. However, in practice, rheumatologists are already using US intuitively to guide the management of these patients; if US synovitis is identified, clinicians often consider starting treatment rather than monitoring for progression [8]. Clearly, there is a significant risk of overtreatment with this approach as many of these symptomatic at-risk patients may not go on to develop clinical synovitis, especially within the short term. Furthermore, if treatment is initiated on the basis of the US findings, there may be little consideration given to the therapeutic regimen and patients could become committed to long-term drugs that might not necessarily be required.

In addition, it is also not clear which joints, and indeed how many joints, need to be imaged for optimum predictive accuracy. Studies in at-risk cohorts have used comprehensive US protocols which include most or all relevant small joints [16, 17]. Although feasible in a research setting, this is time-consuming and not practical in most clinical scenarios. There will need to be further research in identifying the optimum number and distribution of joints and tendons required for risk prediction. Interestingly, a very recent study has demonstrated that the detection of bone erosions in the classic sites for RA damage, especially in the fifth metatarsophalangeal joints, improves prediction of inflammatory arthritis in CCP+ at-risk individuals [14•]. In this context, it would be important to determine if some of the US scores which are routinely used in patients with established disease, such as the US7 or US12, could provide equal diagnostic performances in patients at risk without clinical synovitis [22, 23].

### Ultrasound in the Confirmation of Diagnosis of Rheumatoid Arthritis

The diagnosis of RA is mainly clinically based with many clinicians relying on the fulfilment of the 2010 ACR/EULAR classification criteria for RA [24] for reassurance. The presence of synovitis and RA-related bone erosions are important components of these criteria. Both these EULAR/ACR criteria and the EULAR recommendations for the management of early RA [25] acknowledge the potential value of additional imaging (other than X-ray), such as US, to confirm the presence of inflammation. However, the ACR/EULAR RA criteria state that the information gained from the US can only be used if clinical synovitis has been confirmed in at least one joint, making it problematic for the ‘pre-RA’ group with no clinical findings.

The value of US for the diagnosis of RA lies in its ability to confirm the presence and extent of inflammation and its sequelae in addition to finding alternative explanations for symptoms through a differential diagnosis. One of the most common and challenging subsets of patients are those with undifferentiated arthritis (UA), and especially those that are seronegative. In the context of ‘treat-to-target’ (T2T) strategy, the early identification of patients with UA who will eventually develop RA is of utmost importance to guide early and aggressive therapy during the ‘window of opportunity’ [26]. We will highlight a number of studies that demonstrated that US-proven joint or tendon inflammation might have an important prognostic role for persistent disease in patients with early UA, in which a definite diagnosis cannot be reached.

Freeston et al. evaluated the value of PD signal, in combination to routine clinical management, for the prediction of persistent arthritis in 49 patients with early inflammatory symptoms (early morning stiffness ≥ 1 h in the hands, with or without clinical synovitis, lasting less than 3 months) [27]. At 12 months, 47% of patients developed RA, 31% had other IA (i.e., reactive arthritis or connective tissue disease), and 22% did not develop persistent arthritis. In the patients who were seronegative for rheumatoid factor (RF) and anti-CCP antibodies, but who had high C-reactive protein (CRP), swollen joint count, or bone erosion on conventional radiography, the presence of grey scale (GS) or PD synovitis increased the probability to develop persistent arthritis from 30 to 94%. In a prospective observational study conducted on 60 patients with new-onset UA not fulfilling the 2010 ACR/EULAR RA classification criteria, the presence of GS synovitis at baseline, especially if higher than grade 2, was predictive of progression to RA and methotrexate (MTX) use [28]. Such predictive value was independent of other clinical measures, such as the swollen joint count, or disease activity scores. Interestingly, PD signal was not associated with any of the outcomes evaluated (i.e., progression to RA or MTX use), probably because of the low number of joints showing PD at baseline. In a study by Sahbudin et al., the predictive value of US-detected tenosynovitis and synovitis for the fulfilment the 2010 ACR/EULAR RA classification criteria was evaluated in a cohort of 107 early arthritis patients with clinical synovitis and symptom duration ≤ 3 months [10]. In this study, US-detected tenosynovitis of the finger flexor tendons resulted an independent factor for the prediction of RA, over and above the presence of anti-CCP antibodies and synovitis on US.

### Ultrasound in the Differential Diagnosis

In daily clinical practice, the clinical question that often comes along with a request for an US exam is very simple: ‘Is there any inflammation?’ Although this may appear a reductive concept for using US, it is in reality extremely useful as it allows the immediate differentiation of a potentially serious inflammatory disease from a less serious (in joint terms) mechanical or degenerative process, or unspecified pain syndrome. It should be noted that the interpretation of any US-detected joint inflammatory lesion should be construed in the context of other joint findings. For example, it is acknowledged that inflammation accompanies structural changes of osteoarthritis (OA) in the hands and feet [29]. Once the diagnosis of synovitis (or tenosynovitis) has been established, US may then offer a potential role in the differential diagnosis between the different types of arthritis. In a study carried out by Gutierrez et al., the value of US in the differential diagnosis between RA and psoriatic arthritis (PsA) was evaluated [30]; here in 18 patients with RA and 20 patients with PsA, the presence of peritenon finger extensor tendon inflammation was found in 54 out 82 metacarpophalangeal (MCP) joints in patients with PsA, and in none of the MCP joints of the patients with RA (*p* < 0.001). The results of this study suggest that the presence of inflammation on US at this specific anatomical site is a higher characteristic of PsA and is potentially useful in the differential diagnosis between RA and PsA at the MCP joint level.

The identification of extra-capsular inflammation on US, with or without synovitis, and peri-tendonitis of the finger extensor tendons have also shown to have potential value for differentiating RA from other rheumatic conditions, such as palindromic rheumatism (PR) [31•, 32] and systemic lupus erythematosus (SLE) [33–35]. A significant proportion of patients with PR will eventually develop RA. US evaluation of these patients shows a high prevalence of extra-capsular inflammation (including tenosynovitis, peri-articular inflammation, and peri-tendonitis) during flares [31, 32], with isolated extra-capsular inflammation a specific finding in PR [31•]. It is conceivable that, in PR patients, reversible flares of extra-capsular inflammation eventually progress to persistent intra-articular inflammation as RA develops [36]. As such, US may be invaluable in differentiating a patient with new RA from a patient with PR who does not have intra-articular disease. Such distinction is often difficult on clinical grounds alone, yet it is critically important, as the management of the two conditions is very different.

In a small study, Ogura et al. retrospectively investigated the US abnormalities at joint and tendon levels in the hands of 15 treatment-naïve SLE patients and 40 treatment-naïve RA patients [34]. Interestingly, the authors found a high prevalence of tenosynovitis, which was higher in the SLE group (93% versus 65% respectively, *p* = 0.045). Moreover, it was shown that, differently from what it was observed in patients with RA, the involvement of the finger extensor tendon (i.e., peri-tendonitis) in patients with SLE was frequently detected in absence of joint synovitis, thus suggesting the potential role of US in depicting different patterns of articular involvement in these two diseases.

The accessory pulley linked to the flexor digitorum tendon has emerged a potential specific target of the musculoskeletal involvement in patients with PsA, especially in those with established disease and a previous history of PsA-related dactylitis [37]. In a recent study including 27 patients with RA and 27 patients with PsA, Tinazzi et al. observed that the accessory pulleys are thickened in subjects with PsA compared with RA, especially in the setting of dactylitis, suggesting a potential pathogenetic role of the pulley in the development of tenosynovitis, as well as in the differential diagnosis between PsA and RA [38]. Bone erosions have traditionally been considered as one of the hallmarks of RA, despite not specific for the disease. Interestingly, in a study carried out by Zayat et al. including a total of 310 patients (70 RA, 60 PsA, 60 gout, 60 OA, and 60 healthy volunteers), US has demonstrated the discriminative ability to differentiate RA from other diseases, when large erosions in certain target sites are evaluated [39]. In this study, the presence of larger erosions in selected joints, such as the second and fifth MCP joints, the distal ulna, and the fifth metatarsophalangeal joint, were highly specific for and predictive of RA.

**Fig. 4:**
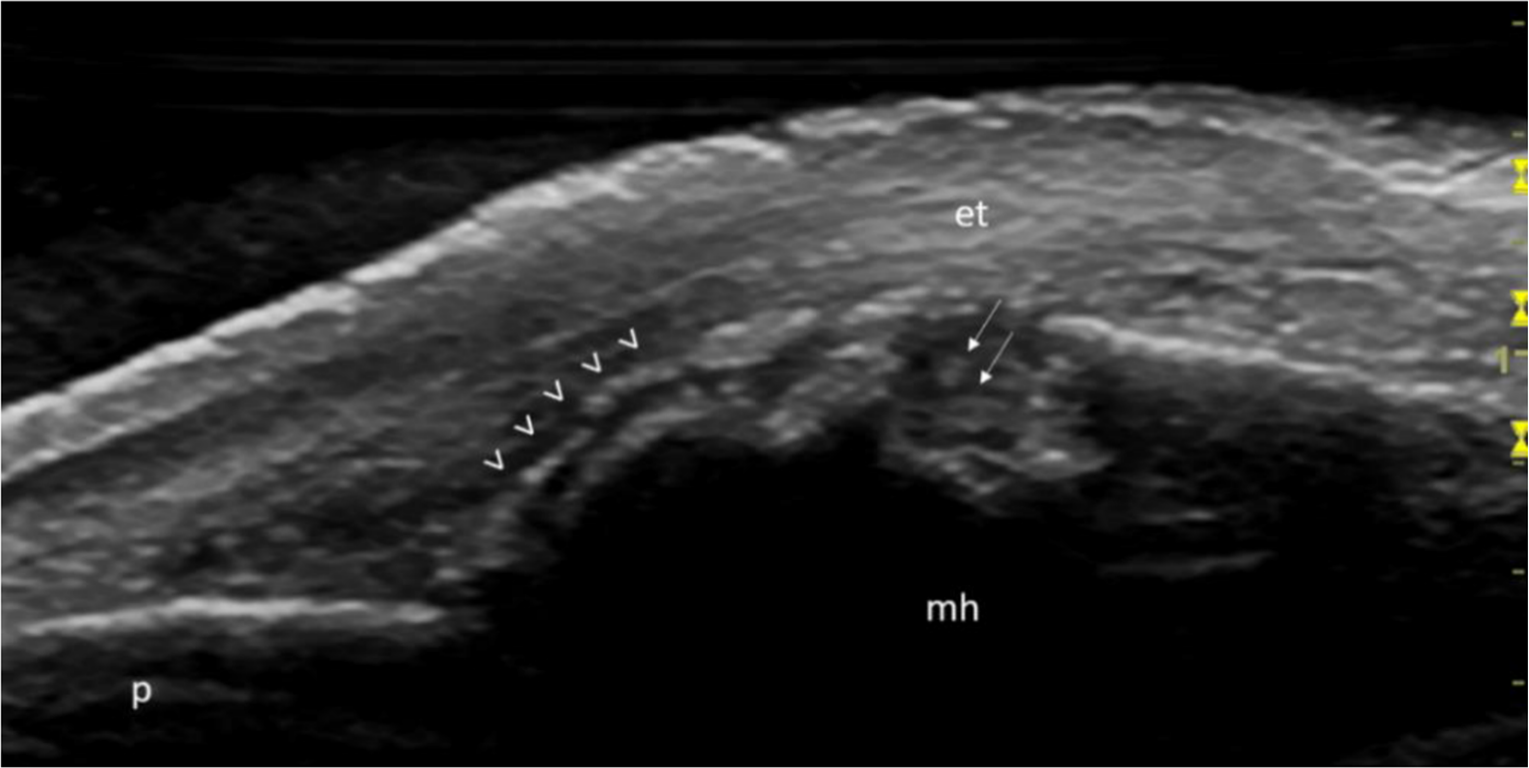
“Double contour sign” and a large bone erosion in the second metacarpophalangeal joint in a patient with gout. Longitudinal scan. The US image shows the presence of the “double contour sign” (arrowheads) over the hyaline cartilage, as well as the presence of a large extra-articular bone erosion filled with hyperechoic spots (arrows) indicating monosodium urate crystal deposits. Legend: et, extensor tendon; mh, metacarpal head; p, proximal phalanx

Clinically, it can be challenging to find firm examination and laboratory or radiological evidence for a crystal arthropathy, even though a clinical history may be suggestive. US has recently begun to be accepted as a diagnostic tool in gout and calcium pyrophosphate deposition disease (CPPD). In these conditions, the spectrum of soft tissues US findings indicating monosodium urate (MSU) or calcium pyrophosphate (CCP) crystal deposits is broad and heterogeneous [40, 41]. Indeed, the ‘double contour sign’, defined by the latest Outcome Measures in Rheumatology (OMERACT) definition as ‘an abnormal hyperechoic band over the superficial margin of the articular hyaline cartilage, which may be either irregular or regular, continuous, or intermittent’, is now an integral part of the rheumatology glossary [42]. It represents the most representative US finding in patients with gout, and it has been included in the latest gout ACR/EULAR classification criteria [43]. However, in patients with suspected gout, performing the US evaluation with the only aim of identifying the ‘double contour sign’ would be limiting, as a wide spectrum of US findings indicating MSU microcrystal aggregates of various size and shape (i.e., ‘hard tophi’, ‘soft tophi’, ‘uratic clouds’) has been described both at joint and tendon levels (Fig. 4) [44–47].

Similarly, the identification of certain US findings within peculiar target tissues (i.e., CCP aggregates in the meniscal and in the wrist triangular fibrocartilages, or within the articular hyaline cartilage) has demonstrated an excellent sensitivity and specificity for the diagnosis of CPPD [48–51]. In the clinical context of suspected crystal arthropathy, the detection of such US findings provides valuable information which might help the rheumatologist in the differential diagnosis decision-making process. However, the correct interpretation of the US findings in these patients might be more insidious in certain clinical scenarios. The high reflectivity which characterizes the microcrystal aggregates might also be generated by other pathological conditions (i.e., degenerative or mechanical damage) and other potential pitfalls, such as the ‘fluid-cartilage interface’ sign, might mimic the ‘double contour sign’, and lead to misinterpretations of the US findings in the hands of a non-expert sonographer [41]. Care, therefore, is warranted when reporting findings and interpreting reports.

### Ultrasound in the Monitoring of Patients with Rheumatoid Arthritis

Several studies have shown that US is able to demonstrate changes in synovitis and tenosynovitis over time in patients with RA. For example, in a study by Naredo et al. on 42 patients with early RA (joint symptoms < 1 year) who started treatment with conventional disease-modifying anti-rheumatic drugs (c-DMARDs), synovitis on US, defined as a combination of GS and PD signals, improved in accordance with the clinical parameters at 12 months follow-up [52]. Similarly, Filippucci et al. evaluated the US changes induced by therapy with adalimumab (ADA) in the wrists of 24 patients with RA, showing a significant improvement in both the clinical and US findings after 12 weeks of treatment. Of note, there was a significant reduction of PD signal at all follow-up examinations (week 2, week 6, and week 12) [53]. D’Agostino et al., in an open-label, multicentre, single-arm study, evaluated patients with RA not responsive to MTX who received intravenous abatacept for 24 weeks. At week 24, there was a significant improvement in the disease activity indices, as well as in the US findings, as documented by the reduction of PD signal [54].

Other than showing changes induced by systemic treatments, US has also the potential to detect changes induced by local therapy. In a study carried out by Gutierrez et al., 114 patients with RA and tenosynovitis were randomized to receive either a conventional “blind” or US-guided local injection with corticosteroids [55]. In the 60 patients who underwent the US-guided injection, the scores of PD, as well as the clinical measures (Health Assessment Questionnaire and Visual Analogical Scale for global and local pain), decreased significantly in the follow-up (4 weeks).

Given that US is able to detect changes in inflammation levels, which patients would most benefit from a scan, given the limitations of resources including time and cost? At present, we consider three scenarios:

1. Patients with long-standing disease who develop new symptoms. Do these relate to active disease, complications of the disease, or a new additional problem?

(2) Patients not responding to therapy. Is the primary diagnosis correct before we consider a switch or escalation in therapy? For example, if US detects no features of RA, this may allow the clinician to consider an alternative diagnosis. The finding of no abnormalities might suggest a chronic pain syndrome whilst the presence of osteophytosis might suggest OA. It should be remembered that the 2010 ACR/EULAR RA classification criteria may generate false positives and from our own data (unpublished), up to 10% of those patients called RA by the criteria, might not actually have the disease.

(3) Patients with significant subclinical disease at baseline which highlighted a substantial mismatch between clinical and US examination. In this scenario, relying on clinical assessment alone may underestimate inflammation load.

### The Utility of Ultrasound in Rheumatoid Arthritis Patients in Clinical Remission

#### Observations on Remission

In comparison to a few decades ago, the prognosis and outcome of patients with RA have improved drastically. Accordingly, the number of RA patients achieving clinical remission has grown exponentially [56, 57]. This appears as the consequence of an earlier diagnoses being made, the application of the T2T strategy following the ACR and EULAR recommendations, and the improvement in the therapeutic armamentarium, with a particular regard to the advent of the biologic (b)-DMARDs [58].

Disease activity in patients with RA is commonly assessed using clinical instruments, such as DAS28-erythrocyte sedimentation rate (ESR) or Clinical Disease Activity Index (CDAI), both in clinical practice and in trials. These composite measures of disease activity rely on surrogate markers of inflammation, such as tender or swollen joint count and inflammatory markers (i.e., ESR, CRP), with the risk of under- or overestimating the disease activity status [59, 60].

#### What Is ‘True Remission’?

Several studies have demonstrated that subclinical inflammation could be found on US in RA patients which are in clinical remission according to the clinical measures (i.e., DAS28-ESR). Naredo et al. explored the prevalence of subclinical synovitis on 67 RA patients in clinical remission (defined as DAS28 < 2.6 or as Simplified Disease Activity Index (SDAI) < 3.3)) which were treated with MTX for at least 2 years [61]. In this study, synovial hypertrophy and PD signal were found respectively in 87.8 and 46.3% of patients in clinical remission according to DAS28, and in 81.8 and in 36.4% of patients in clinical remission according to SDAI. Similarly, in a study including 209 patients with established RA, the presence of subclinical synovitis, as documented by the presence of PD signal, was detected at both 6 and 12 months in the hands of more than 90% of patients in clinical remission after initiation of b-DMARDs [62]. The presence of US-detected subclinical inflammation was found in patients with RA even when more stringent criteria for clinical remission were used. In a study carried out by Brown et al., including 107 RA patients in clinical remission according not only to DAS28, but also to ACR remission criteria and to strict definition of clinical remission (no symptoms and no tender/swollen joints on clinical examination), the prevalence of GS changes and PD signal was very high (73 and 43%, respectively), regardless of the criteria of remission adopted [63].

#### Ultrasound-Detected Subclinical Synovitis in Rheumatoid Arthritis Patients in Clinical Remission: Is it Relevant?

The potential clinical relevance of subclinical synovitis has been highlighted by a few studies which have shown that some RA patients, despite being in clinical remission, do not achieve a good functional outcome and show radiographic progression over time, raising the hypothesis that this could be the consequence of the persistence of such subclinical synovitis [64].

In a longitudinal study, Brown et al. observed that 19% of patients in clinical remission showed radiographic progression at 12 months [65]. Interestingly, the authors demonstrated that the PD scores at baseline were associated with a worse radiographic outcome. Moreover, the presence of PD signal in the MCP joints was significantly associated with radiographic progression in any joint, including the hands and feet. In another study, including 24 patients with established RA (mean disease duration of 114.5 months) in clinical remission, the authors documented a significant association between the presence of subclinical inflammation on US (mainly PD signal) in a particular MCP joint and the presence of bone erosion in that same joint, suggesting a possible link between the presence of subclinical inflammation and the development of bone erosions [66]. In a prospective observational study, including 125 RA patients treated with tumour necrosis factor alfa inhibitors in clinical remission according to DAS28, the authors found that PD signal, especially if greater than grade 1 and near to the bone surface, was significantly associated with radiographic progression at 12 months follow-up [67].

In a very recent prospective study, 383 patients with active moderate to severe RA (CDAI > 10) were managed either with US or according to routine care and followed up for 1 year [68]. In this study, there was no significant difference regarding the clinical outcomes (i.e., CDAI or DAS28-ESR) between the two groups. However, a significant association between PD and GS synovitis at baseline and increased risk for joint damage progression during the follow-up was detected.

These findings have raised the need of a more comprehensive definition on remission, the so called ‘multidimensional remission’, which includes the imaging (and serological) parameters, other than the commonly adopted clinical measures, such as tender or swollen joint count and the physician visual assessments [69]. Longitudinal studies are need to further establish the clinical utility of this new definition of remission.

#### Can Ultrasound Predict the Outcome of Treatment Tapering/Discontinuation in Rheumatoid Arthritis Patients in Clinical Remission?

Given the growing number of patients with RA achieving clinical remission, DMARDs tapering, or even discontinuation, with the aim to reduce costs and safety issues, has now become a ‘hot topic’ and a question of paramount importance [70]. Data from randomized controlled trials (RCTs), registry-based, and observational studies suggest that a status of treatment-free clinical remission is an obtainable target in some patients with RA [71]. On the other hand, other studies have demonstrated that a considerable number of RA patients flare when the therapy is tapered or discontinued, with significant impact of quality of life and possible joint damage progression [72]. In this context, the identification of biomarkers, which might help at delineating the ‘ideal’ patient for treatment tapering or discontinuation, predicting the outcome of such decision, becomes extremely important.

A few studies have demonstrated that the presence of US subclinical synovitis in RA patients in clinical remission might represent a predictive factor for disease flare after treatment tapering/discontinuation. In a study including 42 RA in clinical remission in which treatment with b-DMARDs was discontinued, not the clinical measures (including DAS28), but the presence of US subclinical inflammation at baseline predicted disease relapse following treatment discontinuation [73]. In a study carried out by El Miedany et al., 126 out of 157 patients with RA in clinical remission receiving c-DMARDs and/or b-DMARDs were randomly allocated between 4 different tapering regimes, whereas 31 patients continued the full therapy dose [74]. In this study, the rates of disease relapse were significantly associated with high baseline US scores (both GS and PD scores).

As well as synovitis, also bone erosions have been associated with disease flare in RA patients in clinical remission in which the treatment was tapered or discontinued. In an observational study including 44 RA patients in clinical remission which discontinued b-DMARD therapy, bone erosions on US were an independent prognostic factor for disease relapse 12 months after treatment discontinuation [75]. In this study, neither the presence of US synovitis, nor the clinical or other serological biomarkers (i.e., inflammatory markers), were associated with disease flare at follow-up.

#### Can Ultrasound Help Guide Treatment in Rheumatoid Arthritis Patients?

Despite a growing evidence supporting the potential role of US in the monitoring of patients with RA, including those in clinical remission, the use of US in this clinical context is still limited. Two recent RCTs (TASER and ARTIC) have demonstrated that a treatment strategy based on the US assessment did not lead to an improved clinical outcome in comparison with a conventional T2T approach, suggesting that the systematic use of US in the follow-up of RA patients would be not justified [76, 77].

However, it has been suggested that the methodological design of these studies impacts on their ability to show any difference [78•]. For example, it may be argued that the treatments offered in both arms were already optimized and thus by adding US in was unlikely to show any additional difference. Practically, the offered treatment strategies may also be considered more aspirational than those delivered in the real world, and as such US might have been more likely to have had an observed effect in real world management. It has been postulated that if the study population consisted of patients where there was at baseline a significant mismatch between clinical findings and US rather than applying it to all comers, then a difference may have found. This would have been much more representative of how US is actually applied in every practice.

The authors of ARTIC and TASER have clearly highlighted the need for further work before US could be considered for guiding treatment management, including DMARDs de-escalation, in patients with RA [79]. A feasible and standardized US protocol, which could potentially be integrated into a clinical-centred monitoring strategy, is the ‘conditio sine qua non’ to improve the reliability and clinical usefulness of US in the follow-up of patients with RA. To date, how many joints (and which joints) should be included, which pathological findings should be taken into account, how the US assessment should be carried out (dorsal, lateral, or volar scan), and how to score the US images remain important open questions.

Moreover, more efforts are needed to overcome the intrinsic and well-known limitations of US, such as the high operator-dependence and the consequent large inter-operator variability. Finally, further longitudinal studies are necessary to validate the possible predictive role of the US findings (i.e., subclinical inflammation or bone erosions) for successful (or unsuccessful) DMARDs tapering or discontinuation in RA patients in clinical remission.

## Conclusions

This review has highlighted the current and potential utility of US imaging across the RA continuum. Its ability to identify early inflammatory and structural changes in joints and soft tissues has clear benefits for early diagnosis and prediction of outcome through risk stratification and ensuring optimal disease control. As technology improves, US is allowing new insights into understanding joint pathology as well as helping differentiation between diseases. As with all research, as more data is produced, more questions are being asked. More appropriately designed RCTs are required to identify which groups of patients benefit the most from US imaging.
